# Multifaceted Pathophysiology and Secondary Complications of Chronic Spinal Cord Injury: Focus on Pressure Injury

**DOI:** 10.3390/jcm14051556

**Published:** 2025-02-26

**Authors:** Mario Martínez-Torija, Pedro F. Esteban, Angela Santos-De-La-Mata, Matilde Castillo-Hermoso, Eduardo Molina-Holgado, Rafael Moreno-Luna

**Affiliations:** 1 Pathophysiology and Regenerative Medicine Group, Hospital Nacional de Parapléjicos, SESCAM, 45071 Toledo, Spain; mariomartineztorija@gmail.com (M.M.-T.); asantosde@externas.sescam.jccm.es (A.S.-D.-L.-M.); mcastilloh@sescam.jccm.es (M.C.-H.); 2 Pathophysiology and Regenerative Medicine, Instituto de Investigación Sanitaria de Castilla-La Mancha (IDISCAM), 45004 Toledo, Spain; 3 Department of Nursing, Hospital Universitario de Toledo, SESCAM, 45071 Toledo, Spain; 4 Grupo de Neuroinflamación, Hospital Nacional de Parapléjicos, SESCAM, 45071 Toledo, Spain; teban@sescam.jccm.es (P.F.E.); eduardom@sescam.jccm.es (E.M.-H.); 5 Unit of Internal Medicine and Intermediate Respiratory Care, Hospital Nacional de Parapléjicos, SESCAM, 45071 Toledo, Spain; 6 Grupo de Neuroinflamación, Instituto de Investigación Sanitaria de Castilla-La Mancha (IDISCAM), 45004 Toledo, Spain

**Keywords:** spinal cord injury, wound healing, adipose tissue, pressure injury

## Abstract

**Background/Objectives**: Spinal cord injury (SCI) is a complex medical condition with widespread effects that extend beyond motor and sensory impairments. In addition to nervous system damage, SCI patients experience various secondary complications, including vascular dysfunction, altered body composition, and metabolic disturbances. Among the most common secondary pathologies is the development of pressure injuries (PIs), chronic wounds that significantly affect quality of life and can be challenging to treat. Understanding the physiological and cellular mechanisms behind these complications is crucial for improving care and therapeutic outcomes. **Methods**: We conducted a comprehensive literature search in PubMed, Scopus, and Google Scholar using keywords related to spinal cord injury, pressure ulcer/pressure injuries, metabolic and vascular dysfunction, biomechanics, and regenerative therapies. Studies were selected based on their relevance to the pathophysiology, risk factors, and novel therapeutic approaches for PIs in SCI patients. **Results**: Vascular dysfunction, characterized by impaired blood flow and microcirculatory issues, predisposes SCI patients to ischemia and tissue necrosis, particularly in areas subjected to prolonged pressure. Additionally, changes in body composition, such as increased adiposity and muscle atrophy, further compromise tissue integrity and healing capacity. The inflammatory response, mediated by cytokines such as IL-1, IL-6, and TNF-α, exacerbates these effects by sustaining a pro-inflammatory environment that delays the transition of macrophages to the M2 phenotype, critical for wound healing. External factors, such as poor nutrition, infections, and immobility, also play a significant role in worsening the wound healing process. **Conclusions**: Chronic SCI induces a cascade of physiological changes that predispose patients to the development of PIs and complicate their recovery. The intricate interplay of vascular, metabolic, and inflammatory responses creates a hostile environment for wound healing. A deeper understanding of these systemic effects is essential not only for developing targeted therapeutic strategies to improve chronic wound healing but also for refining preventive approaches that minimize their occurrence. Advancing this knowledge will ultimately help enhance the quality of life for individuals with SCI.

## 1. Introduction

Spinal cord injury (SCI) stands as a profound and multifaceted medical condition that reverberates throughout the body, affecting not only the spinal cord itself but also various organs, tissues, and physiological systems [1,2,3]. Beyond the immediate impact on motor and sensory functions, SCI is often accompanied by a range of secondary complications that significantly contribute to the complexity of patient care and overall well-being.

The pathophysiology of SCI extends beyond the initial trauma, involving intricate interactions between cellular and molecular processes that lead to widespread systemic changes. Among the numerous secondary complications experienced by individuals with chronic SCI, vascular dysfunction stands out as a prominent issue [3]. Impaired blood flow and altered vasomotor control not only hinder tissue oxygenation but also predispose patients to various cardiovascular complications, highlighting the systemic impact of SCI beyond the neural realm.

Another critical consequence of chronic SCI is the redistribution of body composition, with the loss of extracellular matrix in regions situated below the level of injury, such as the lower extremities and the gluteal region [4,5]. This loss of structural support contributes to diminished tissue integrity, rendering these areas susceptible to the development of pressure injuries (PIs), also known as pressure ulcers or wounds, a prevalent secondary pathology in individuals with SCI [6,7]. The mechanism of the appearance of PIs has been widely described too. Usually located on bony prominences such as the ischial tuberosity, sacrum, or calcaneus, prolonged pressure on these areas generates a considerably reduced blood flow, causing ischemia and subsequent necrosis, giving rise to wounds that are initially well-defined that, if not healed, can affect not only the deeper layers of the skin but also soft tissues such as muscle, fat or bone [8,9,10]. In this sense, understanding the intricate relationship between tissue matrix changes and loss, and the development of PIs is paramount for devising effective preventative and therapeutic strategies.

External factors further exacerbate the challenges faced by individuals with chronic SCI. Poor dietary habits, sedentary behaviors, and increased susceptibility to infections compound the multifaceted consequences of the injury. Moreover, nutritional deficiencies can impair tissue repair and compromise immune function, while the lack of physical activity accelerates muscle atrophy, bone density loss, and cardiovascular deconditioning [6,7,11,12]. These factors collectively contribute to an increased risk of complications and an overall decreased quality of life for individuals with chronic SCI.

Intriguingly, the pathophysiological cascade following SCI extends its reach to adipose tissue [5], which has evolved from being merely an energy reservoir to a dynamic participant in metabolic regulation and immune responses [13]. The adipose tissue is also recognized as a vital reservoir of stem cells responsible for tissue maintenance and repair [14,15]. These cells, with their potential for differentiation into various cell lineages, play a crucial role in tissue regeneration and healing processes. Alterations in the composition and function of adipose tissue post-SCI could potentially lead not only to metabolic shifts but also to the deterioration of tissue integrity, thereby exacerbating the risk of secondary complications.

This narrative review aims to analyze the internal and external factors involved in the pathophysiology of SCI, emphasizing that the onset and progression of PIs in individuals with SCI are not solely the result of inadequate management of prolonged pressure on the tissues. While sustained pressure is a key factor, it is essential to recognize the broader range of underlying pathophysiological mechanisms, including vascular dysfunction, autonomic dysregulation, and metabolic changes, which not only increase susceptibility to PIs but also interfere with their progression and healing. Understanding these processes is crucial for optimizing preventive and therapeutic strategies that extend beyond simple pressure relief.

## 2. Materials and Methods

The present paper consists of a narrative review whose objective is to know the internal and external factors related to SCI that predispose to the development of PIs. A search was conducted in PubMed, Scopus, and Google Scholar databases from their inception to the present. The following search terms were used: “spinal cord injury”, “pressure ulcer/pressure injuries”, “wound healing”, “pathophysiology”, “subcutaneous tissue”, “connective tissue”, and “impaired healing”. All the major journals were indexed. Articles without the full text electronically available were also excluded. After removing the majority of papers not focusing on the changes occurring after SCI, and its relationship with PI formation and/or impaired wound healing, we considered the articles referenced in the present review.

## 3. Results

SCI generates a clinical condition that entails a series of significant body changes, extending beyond the characteristic motor and sensory impairment. Among them, alterations in body composition, metabolism, and inflammatory response stand out. These systemic changes significantly contribute to the pathophysiology of PIs, making SCI patients highly susceptible to their development. Specifically, they undergo qualitative and quantitative modifications in bone density, as well as in muscular and adipose tissue; factors that, combined with metabolic changes and hormonal imbalances, increase vulnerability to the onset of PIs.

### 3.1. Nervous System and the Biomechanical Implications of Immobility in PIs Development

To understand the modifications that occur in connective tissue, it is essential to know the repercussions on the peripheral nervous system underlying SCI. Progressive changes between the acute and chronic phases of SCI have been broadly studied: after an initial period of residual hyperactivity, the nervous system decreases its activity during a prolonged acute phase with signs of parasympathomimetic activity and absence of reflexes below the level of injury [16]; finally, a state of autonomic dysreflexia characterizes chronic SCI, especially in patients with injury at T6 or above, with hyperreflexia and motor spasticity [17].

As mentioned previously, SCI results in total or partial loss of motor function in the patient. The loss of function and muscle atrophy is primarily, although not solely, due to damage originating in the somatic nervous system: upon interruption of electrical conduction, a significant amount of motor neurons directly undergo apoptosis; the remaining neurons undergo modifications in both caudal and rostral ends [18], negatively affecting synapses and neuromuscular connection [19]. Additionally, SCI also involves damage to the functioning of the autonomic portion of the peripheral nervous system. The organization of the autonomic nervous system into two well-defined axes, sympathetic and parasympathetic, as well as its involvement in bodily homeostasis, involuntarily regulating the activity of peripheral organs in higher organisms, has been widely described [3].

In this context, several biomechanical factors contribute to the heightened susceptibility to PIs in SCI patients. The combination of motor paralysis and neuromuscular atrophy significantly restricts mobility, leading to prolonged and unrelieved pressure over bony prominences. The sustained compression of soft tissues exceeds capillary perfusion pressure, causing vascular occlusion, diminished oxygenation, and progressive ischemic damage, ultimately leading to tissue necrosis. The absence of sensory feedback further exacerbates the condition, as individuals with SCI cannot perceive pressure-induced discomfort, delaying necessary repositioning and increasing tissue breakdown severity [20].

Moreover, autonomic dysfunction impairs the body’s ability to regulate blood flow in response to mechanical stress. In the acute phase, parasympathetic overactivity reduces vascular tone and impairs vasodilation, intensifying tissue hypoxia. In the chronic phase, patients with lesions above T6 frequently experience autonomic dysreflexia, which manifests as episodic hypertensive crises. When combined with motor spasticity and hyperreflexia, these hemodynamic fluctuations amplify friction and shear forces within tissues, further exacerbating tissue vulnerability. Shear stress occurs when adjacent tissue layers shift in opposing directions, damaging cellular structures and small blood vessels, worsening ischemia, and accelerating PIs formation.

Furthermore, muscle atrophy diminishes the protective function of soft tissues, increasing direct pressure on skeletal prominences and further heightening PIs risk. The combined effects of decreased muscular support, impaired vascular response, and disrupted sensory feedback create an environment in which even minor mechanical stressors can lead to severe tissue breakdown.

Taken together, these factors highlight the intricate relationship between neurological dysfunction, biomechanical stressors, and compromised vascular responses in SCI patients. However, beyond the direct mechanical implications, SCI-induced PIs must be understood as a consequence of broader systemic alterations that extend beyond immobility and sensory loss. The chronic nature of SCI leads to progressive dysfunction across multiple physiological systems, each contributing to the development, persistence, and severity of PIs [21]. An overview of the most relevant pathophysiological changes involved in the development and progression of PIs in patients with SCI is outlined in Table 1.

### 3.2. Vascular System

Among the targets of the sympathetic nervous system is vascular tissue. As previously discussed, the chronic condition of autonomic dysreflexia in SCI patients involves a high level of circulating catecholamines causing a vasoconstrictive response [22], especially in those individuals with lesions above T5. Additionally, tetraplegic patients have shown a higher number of abnormal vascular events (severe hypertension, orthostatic hypotension, etc.) compared to paraplegics [23]. Furthermore, the neuroinflammatory state underlying SCI leads to the release of soluble tumor necrosis factor-alpha (sTNFα), further contributing to autonomic dysreflexia and vascular tissue dysfunction [24]. These changes in vascular innervation have been linked to a significant reduction in vessel caliber [25,26,27] as well as blood flow [27,28]; moreover, even the vessel’s own reactivity, the capability to self-regulate its tone in response to stimuli, is also impaired, especially in individuals who are more sedentary or have a higher body mass index [29].

A situation of low perfusion and ischemia leads to the formation of reactive oxygen species (ROS), which exert an oxidative effect on the tissue and hinder its regeneration [30]. While it is true that intrinsic changes due to SCI generate such a hypoxic environment, improper loading on bony prominences further contributes to vessel and capillary occlusion, favoring the main mechanism of PIs formation [31]. Furthermore, when postural changes are not made properly, tissue reperfusion generates a large amount of ROS, damaging additionally the tissue [32].

Endothelial cells, responsible for forming the vessel bed, also appear to undergo changes, namely, increased cytoplasmic activity, a highly rich content of free ribosomes and mitochondria, or a thicker-than-usual basement membrane, among others. On the other hand, pericytes have scant cytoplasm but are occupied by numerous micropinocytic vesicles [33].

Finally, the role of microcirculation as a mechanism that specifically allows the exchange between blood and tissue is well recognized. In fact, microcirculation increases at the center of ulcers, supporting their recovery [34].

Some studies with SCI patients claim the existence of changes where most capillaries appear occluded [31,33]. Even more, a recent review by Benitez–Albiter and colleagues [35] not only documents a significant number of endothelial changes in the microcirculation following SCI but also suggests a connection between larger caliber vessels and the high prevalence of cardiovascular pathologies in this patient population.

This persistent dysregulation of the vascular system highly increases the risk of developing PIs.

### 3.3. Adipose Tissue

The classic paradigm has regarded adipose tissue as a reservoir of energy. However, its capability to store large amounts of lipids and release them according to energy demands, as well as its aptitude to produce regulatory adipokines, has transformed adipose tissue into an organ with recognized endocrine-metabolic functions [36].

Moreover, fat harbors stem and progenitor cells capable of maintaining connective tissue homeostasis and enabling tissue repair. Adipocytes and immune cells coexist with mesenchymal stem cells residing in adipose tissue (AT-MSCs), which can differentiate into various cell types, such as adipocytes, osteocytes, myocytes, or chondrocytes [14,37]. While they do present a specific combination of markers allowing their isolation (CD34+/CD31−/CD45−/CD90+), recent studies have described the presence of new phenotypes defining different subpopulations responsible for specific functions [38], which could help better understand the role of adipose tissue in tissue regeneration. Additionally, there exists a subtype of progenitor cells originating from the vast network of vessels that cross and nourish adipose tissue: endothelial colony-forming cells (ECFCs), which combine exceptional clonal ability and expansion potential with a robust capacity to form blood vessels [15].

However, the sustained imbalance between consumed and required calories, along with the proinflammatory role of immune cells and cytokines in adipose tissue, could lead to its dysfunction, termed as adiposopathy, disrupting endocrine-metabolic homeostasis and tissue regeneration capacity [37].

At the macroscopic level, SCI leads to increased adipose tissue and consequent obesity as a common phenomenon [39,40]. Apparently, no differences are observed in the thickness of subcutaneous tissue between patients with and without SCI [20]; however, these changes are particularly emphasized in the accumulation of visceral fat tissue [39,41], lean muscle tissue [5,42,43], and even bone marrow [44]. Schwartz et al. [42] correlated intramuscular fat accumulation with increased fatty acid-binding protein (FABP3 and FABP4) levels, proteins implicated in muscle atrophy, and increased risk of pressure ulcers.

Adiposity has even been linked to the level of injury: individuals with tetraplegia presented higher total and visceral adipose tissue content than those with paraplegia, and among them, the higher the level of injury, the more fat is stored [5]. Due to a higher total amount of adipose tissue, the production of adipokines is strongly linked to the context of SCI. Factors such as increased TNF, IL-6, and other proinflammatory adipokines [40,45,46,47] are present in SCI patients and correlate with the abundance of adipose tissue, playing an essential role in the systemic inflammatory response. Furthermore, their presence has been associated with the release of non-esterified fatty acids (NEFAs), which promote an increase in the production of LDL and VLDL [48]. Additionally, Wang et al. [49] determined an increase in blood levels of leptin and adiponectin, which could predispose these individuals to insulin-resistance development.

Therefore, the changes occurring in adipose tissue as a result of SCI align with the development of PIs. Excess total fat storage contributes to poorer functionality of MSCs, both in adipose tissue itself [50] and in the bone marrow, whose functionality could be altered once infiltrated [4,51], further possibly impairing wound healing if an ulcer appears [50,52]. Furthermore, the underlying low capillary density creates a hypoxic environment, exacerbating vascular insufficiency [53]. Whether this issue is due to increased adiposity or applies to SCI patients remains a question yet to be resolved.

### 3.4. Muscle Tissue

Similar to the increase in total adipose tissue, muscle tissue experiences a decrease in density, indicating a “cross-talk” between both tissues where the first one negatively impacts the second one [4]. In addition to the multiple factors described explaining the subsequent atrophy after SCI, muscular inactivity due to motor loss, as well as dysfunction of the autonomic nervous system, lead to increased proteolysis and tissue degradation [18,19]. As described above, immediately following the injury, a spinal shock occurs, causing severe loss of motor function and sensation as well as a state of hypo- or even absence of reflexes, known as flaccid paralysis, later evolving into a state of spasticity, where exaggerated reflexes appear [54]. A study by Trolle et al. [55] determined that from the onset of the injury, the patient undergoes massive loss of lean tissue, followed by a more progressive loss throughout their life. The level of injury seems to be related to the degree of atrophy in paraplegic patients [56], with certainty that tetraplegics suffer greater muscle loss [57]. In terms of cross-sectional diameter, SCI patients exhibit shorter length and greater atrophy than those without disabilities [58]; however, some patients may present a false increase in total muscle mass due to the aforementioned increase in fatty infiltration [20]. This reduction in total muscle mass is reflected in decreased serum creatinine levels in these individuals [59].

It is not just the overall muscle mass that has changed. Following the injury, around 6 months, a modification of muscle fiber type is observed, with a reduction of type I fibers, commonly known as oxidative, towards highly glycolytic type IIb fibers [60]. Moore et al. [58] detected a significant reduction in muscle tissue in patients with more than two years of SCI.

At the molecular level, factors such as TNF-α [61] or TNF-related apoptosis-inducing ligand/factor (TWEAK); interleukins 1β (Il-1β) [62] and 6 (Il-6) [63]; and other muscle cytokines such as atrogin-1 (Fbox-1), ubiquitin ligase E3 (MuRF1) [64], myostatin [65], or calpain [66] have seen their production modified from the onset of SCI, generating inflammation in muscle tissue, atrophy, and loss of lean mass. Furthermore, the production of insulin-like growth factor type I (IGF-1), directly involved in muscle development, cell differentiation, and collagen production, is decreased in these patients [67,68]; such production, induced in the liver by the presence of growth hormone (GH), would be inhibited precisely by the dysfunction of this hypothalamic-hepatic axis in the concurrence of SCI and GH-resistance [69]. Thus, the inherent atrophy in SCI carries a higher risk of PIs in the patient due not only to the friction and shear forces generated by support surfaces but also to a greater deformation suffered by subcutaneous tissues over bony prominences [70,71]; furthermore, the decrease in lean mass correlates directly with arterial dysfunction in SCI patients [25], reinforcing the constant thesis of peripheral ischemia contributing to the development and poor healing of wounds.

### 3.5. Bone Tissue

Osteoporosis is a direct consequence of SCI [72]. The loss of lean mass and decreased tension on the bone are two of the main factors that determine bone tissue deterioration [73]. These patients present a very marked decrease in bone density during the acute phase up to the first year of evolution [74,75], with estimated losses of 2 to 4% per month [76], reaching up to 20% in lower limbs, typically femoral head and hip [76,77]; however, existing literature indicates that such a reduction does not take place in the lumbar region [77,78] nor upper limbs [79]. This loss persists throughout the patient’s life [80,81]; additionally, El-Kotob et al. [82] determined that the loss occurs on the cortical mass of the bone, not in the trabecular area; furthermore, Abdelrahman et al. [74] affirm that cortical changes vary regionally depending on the studied point of the bone cortex; on the other hand, Gorgey et al. [44] found that the decrease in cortical thickness was associated with an increase in yellow marrow, which agrees with the aforementioned comments regarding bone marrow fat infiltration and decreased osteocyte production [83]. In turn, individuals with tetraplegia also experienced a greater decrease in bone density than paraplegic ones [78,81].

Bone density not only varies following SCI. Changes in geometry and mineral distribution in the cortical layer of the bone are also observed, causing a decrease in the torsional strength of load-bearing structures, mainly the femur and tibia [76,80], significantly increasing the risk of fracture in these patients. Regarding the loads supported by osteocytes in the bone matrix and how these vary in the context of SCI, the production of molecules of the mechanotransduction system may be modified. One of the most representative is sclerostin, responsible for inhibiting the Wnt/β-catenin pathway, favoring bone resorption. In a recent study by Li et al. [84], they used a biopolymer (S8 + BBR FFPS) capable of activating the Wnt/β-catenin pathway to improve chronic wound healing; therefore, high levels of sclerostin due to SCI could hinder wound healing. However, there seems to be a discrepancy between studies, with some finding SCI patients with decreased [85] or elevated levels [65] of this molecule.

Serum levels of periostin, involved in osteoblast development and bone matrix formation, also do not seem to correlate with fracture risk in SCI individuals [85]. Furthermore, the study of other markers involved in bone metabolism, such as osteocalcin [86,87] or C-telopeptide [86], as well as their relationship with the level or degree of injury [87], do not provide clear results on their influence.

Additionally, vitamin D deficiency, a hormone involved in skeletal remodeling and maintenance of optimal bone density, has been widely described in these individuals [59,88]. Consequently, this correlates with elevated levels of parathyroid hormone [88], which contributes to the osteoporotic status of the patient and increased fracture risk.

However, one of the less frequent and most striking phenomena in the musculoskeletal system is the formation of bone in soft tissues, known as heterotopic ossification (HO). This event, found at the level of the hip and knee—primarily but not exclusively [89]—increases the high-pressure points to which subcutaneous tissue is subjected and further predisposes to the development of PIs [90]. Although the mechanism is not fully understood, factors such as alkaline phosphatase [91] and interleukin 1β (Il1β) [92] seem to be closely related. A meta-analysis published by Yolcu et al. [89] determined that among the factors related to HO in SCI individuals are a higher degree of injury, male sex, smoking, spasticity (related to the release of Il1β at the muscular level), and the presence of PIs. The fact that PIs contribute to the occurrence of HO and, in turn, perpetuate these chronic wounds, creates a vicious circle that makes their treatment essential.

### 3.6. The Immune System and Skin Microbiome

The risk of infection is significantly increased in patients with SCI. In addition to the overall response that occurs following the SCI, the low-grade systemic inflammation coincides with a paradoxical state of spinal cord injury-induced immune depression syndrome (SCI-IDS) [93,94]. Valido et al. [95] summarized in three points why this state occurs in patients with SCI: first, due to the loss of neuroendocrine innervation of peripheral organs such as the spleen, adrenal glands, and bone marrow; second, the aforementioned alteration of autonomic innervation; and finally, how permeability changes in certain membranes lead to gut microbiota alterations in SCI patients.

Throughout the review, multiple studies have been presented that document cytokine production in various tissues and organs from the early days following SCI [4,18,19,48,93]. While the presence of these mediators is necessary to facilitate the immune response, their persistence over time results in alterations in the hypothalamic-pituitary-adrenal axis, overstimulating the adrenal gland and the ulterior uncontrolled production of glucocorticoids, suppressing maturation, differentiation, and proliferation of immune cells, and leading to adrenal gland exhaustion, with the subsequent systemic immunosuppression [93,96]. This is evidenced by the expression and detection of markers such as CRP, TNF-α, IL-2, and IL-6, among others, which are increased in the sera of patients after several weeks of progression [47,95,97]. Additionally, Bloom et al. [98] noted differences in cytokine detection between the acute and chronic phases.

In addition to the associated increase in cytokines, a change in the cellular profile of these patients can also be observed. In general terms, more leukocytes appear with a sudden and significant increase in monocytes and neutrophils, although lymphocytes decrease, primarily reflecting the systemic inflammation that follows the establishment of SCI [95]. However, over the weeks, changes in cell number and activity occur, further contributing to the poor immune response of the patient [95]. During the chronic phase, a decrease in the phagocytic capacity of neutrophils [99] and monocytes [100] is observed, as well as a reduction in the cytotoxic activity of T lymphocytes and a diminished Th1 response in favor of Th2 [96]. Additionally, a diminution in the number of NK cells and dendritic cells is recorded [101], along with the inability of macrophages to switch from a pro-inflammatory (M1) to an anti-inflammatory (M2) phenotype, directly impairing wound remodeling and healing [62]. Furthermore, the presence of a subpopulation of CD74+ B lymphocytes may explain the autoimmune component observed in these patients [102].

The severity of the injury also influences the immune system. Thus, individuals with complete injuries are more likely to suffer damage to the sympathetic plexus, negatively affecting how the bone marrow controls hematopoietic progenitors [103]. Furthermore, SP1 transcription factor levels, involved in the early stages of hematopoietic differentiation, seem to be decreased [101]. Several studies have demonstrated that hematopoietic cells play a central role in vascular morphogenesis [104,105], so their alteration could further contribute to poor maintenance and/or repair of vascular structures in these patients. Besides white cell damage, the presence of central-origin anemia has also been described, independent of the nutritional status of the patient [106].

Other changes, such as alterations in the microbiota [107] or lymphoid tissue [30], vitamin D deficiency [88], cortisol elevation [93,108], or even differential expression of chemokines and other adhesion molecules [108,109,110], may contribute to the poor systemic immune status of these patients, increasing the risk of infection.

On the other hand, due to the exposure of the skin to pathogenic agents and the harmful role they can play in wound healing, infection prevention is essential. Although the skin microbiome does not seem to undergo intrinsic changes after SCI, Dana et al. [111] pointed to a higher presence of gram-negative bacteria in PIs due to urinary incontinence, as well as methicillin-resistant *Staphylococcus aureus* (MRSA) due to prolonged hospital stays. Then again, Dunyach-Remy et al. [112] associate one or other bacterial genre with better or worse healing, not only due to the pathogenic but also to the symbiotic nature that some microorganisms may present. Traditionally, bacterial infections, both local [113] and systemic [114], have been associated with delayed healing by promoting a pro-inflammatory environment that prevents macrophages from adopting a phenotype capable of facilitating matrix remodeling [62]. Moreover, lipopolysaccharides, which belong to the Gram-negative bacteria envelope, link to MSCs via toll-like receptor 4 (TLR4), altering the functionality of these cells and leading to differentiation toward HO [115], which could further slow down wound healing [116]. Finally, not only do bacteria seem to exert an effect on wound healing mechanisms; recent studies have associated COVID-19 infection with a prolonged inflammatory state, which gives the impression of having a direct impact on the mechanisms of chronic wound healing in SCI patients [117].

### 3.7. Endocrine System and Metabolism

The change in the overall body composition of SCI patients not only affects the proportion of muscle and adipose tissue but also influences the total energy expenditure. Muscle atrophy and fat deposition, coupled with a significantly more sedentary lifestyle, lead to a noticeable decrease in energy requirements. In general, living a sedentary life entails an energy surplus and increased adiposity [118]. Subsequently, this not only affects the individual’s body composition but also predisposes them to a series of endocrine changes that increase their morbidity and mortality, primarily highlighting three main aspects: a peculiar pattern of dyslipidemia characterized by very low levels of HDL [48]; overexpression of proinflammatory adipokines related to vasoconstriction and hypercoagulability [48]; and insulin resistance [119,120], directly linked to reduced activity of the PI3K pathway due to the accumulation of obesity-derived proinflammatory cytokines [67]. Controversially, and regarding this last point, SCI does not seem to significantly increase the prevalence of diabetes mellitus compared to non-injured obese individuals [59,121]. Nevertheless, a higher prevalence of metabolic syndrome in these patients, along with the development of hypertension and atherosclerosis, leads to an increased cardiovascular risk [122,123].

Two axes, the hypothalamus-pituitary-adrenal and the hypothalamus-GH-IGF1, are affected. As mentioned earlier, alteration of the hypothalamus-pituitary-adrenal axis leads to a meager regulation of circulating glucocorticoids in the body. This, besides interfering with the inflammatory response, generates a state of hyperglycemia and inhibition of protein synthesis, increasing the patient’s risk of diabetes mellitus and muscle atrophy [93]. On the other hand, the production of IGF1 is also impaired due to the aforementioned decrease in hypothalamic PI3K activity [67]. This kinase is essential for maintaining muscle tissue, promoting cell proliferation and differentiation, collagen formation, and ultimately wound healing. Additionally, its lack of production significantly decreases muscle anabolism, leading to atrophy [69]. Other hormones involved in tissue regeneration, such as testosterone [59] or the previously mentioned vitamin D [88], have their production inhibited.

The decrease in the activity of the mitochondria is particularly noticeable. Due to the long periods these individuals spend immobilized, glycolytic and oxidative pathways exhibit much lower activity, resulting in decreased ATP production via oxidative phosphorylation and increased ROS [124].

Overall, all these changes result in an environment characterized by low perfusion, the presence of proinflammatory elements, the tendency to develop metabolic syndrome, and difficulty in anabolic processes, compromising thus the reconstruction of damaged tissue.

### 3.8. Preventive and Therapeutic Strategies for Pressure Ulcers in Patients with Spinal Cord Injury

At this point, it is evident that wound development and subsequent healing in patients with SCI is a complex and multifaceted process influenced by both intrinsic and extrinsic factors. While traditional wound care approaches emphasize nutritional support, infection control, and pressure relief, these measures often fail to fully address the underlying pathophysiological alterations that impair tissue repair. A deeper understanding of how vascular dysfunction, metabolic imbalances, and regenerative deficits interact in patients with SCI is essential for developing targeted therapeutic interventions that go beyond conventional wound management.

#### 3.8.1. Nutrition

Nutrition plays a fundamental role in overall health and in preventing complications in individuals with chronic SCI [125]. A well-equilibrated diet not only helps maintain an appropriate weight but is also essential for wound healing, infection prevention, and skin integrity maintenance [126,127]. However, individuals with chronic SCI face unique challenges that may limit the effectiveness of standard nutritional interventions.

Alterations in body composition, characterized by decreased muscle mass [4,55,56] and increased fat mass [39,40,41], lead to a reduction in basal energy expenditure [118]. This decline in caloric requirements [118], combined with reduced mobility [4,57], can contribute to overweight and obesity if caloric intake is not properly adjusted [39,41]. Additionally, the loss of muscle mass [4,55,56] results in a diminished protein reserve, which can hinder wound healing [67,68] and increase the risk of developing PIs [70,71].

Since malnutrition is common in patients with PIs and represents a significant risk factor for delayed healing, nutritional assessment should be a priority in this population. In 2012, a specific tool for evaluating the nutritional status of SCI patients, the Spinal Nutrition Screening Tool (SNST), was validated [128]. Although the Malnutrition Universal Screening Tool (MUST) remains widely used in most healthcare centers. Nutritional intervention throughout the entire prevention and treatment process is a fundamental pillar in patient management across all settings [129]. Even so, despite the existing knowledge, further research is essential to gain a deeper understanding of the specific nutritional needs of this population and to develop more effective interventions [129,130]. The high risk of malnutrition in SCI patients is associated with negative impacts on rehabilitation, delayed wound healing, increased infection rates, and a higher prevalence of pulmonary diseases, among other complications [131].

In terms of nutritional interventions, protein, arginine, zinc, and vitamins C and A supplementation have been extensively documented as facilitators of wound healing mechanisms in SCI patients [108,132]. A protein intake of 1.25 to 1.5 g/kg/day is recommended to optimize tissue regeneration and accelerate PIs healing [133]. However, the response to nutritional therapy may be compromised by multiple additional factors. Metabolic alterations in these patients, including changes in the production and regulation of hormones such as insulin and insulin-like growth factor 1 (IGF-1) [67,68,69], may reduce the efficiency of protein synthesis and tissue repair, even when nutritional intake is adequate. Furthermore, vascular dysfunction [25,27,29] and hypoxia in PI-affected tissues [53,70,71] can limit the absorption and transport of essential nutrients to the wound site, reducing the effectiveness of supplementation.

Beyond these physiological challenges, socioeconomic barriers may also hinder the implementation of adequate nutrition in individuals with SCI [134]. Economic limitations can restrict access to fresh, nutrient-dense foods, while physical barriers may complicate the preparation of healthy meals. A lack of support or assistance with grocery shopping and meal preparation may lead to reliance on processed foods with lower nutritional quality, exacerbating the risks of malnutrition and associated complications.

It is important to recognize that while proper nutrition is essential, it may not be always sufficient to prevent or treat PIs in SCI patients. The presence of comorbidities such as recurrent infections, metabolic disorders, immune dysfunction, and vascular impairments can interfere with healing processes, even when nutritional protocols are followed. Therefore, a multidisciplinary approach that integrates nutritional strategies with medical, social, and economic interventions is crucial to improving outcomes in PIs prevention and treatment within this population.

#### 3.8.2. Mobilization and Repositioning

Therapeutic strategies to prevent PIs in patients with SCI include periodic repositioning and continuous proper body alignment [135]. Although these measures are essential, putting them into practice can be challenging due to the neurological limitations these patients face.

During the acute phase of immobility, a mobilization schedule every 2 to 3 h is recommended to prevent prolonged pressure [135]. However, as discussed in Section 3.2, the loss of motor function, whether partial or complete, can prevent patients from repositioning themselves, making them dependent on constant assistance from healthcare professionals or caregivers. Additionally, muscle atrophy and the activity shift from type I to type IIb muscle fibers reduce natural cushioning over bony prominences, significantly increasing the risk of PIs.

When seated, proper posture and even weight distribution are essential to relieve pressure on vulnerable areas. However, autonomic nervous system dysfunction, particularly in injuries above T6 [17], can lead to autonomic dysreflexia, a condition marked by hyperreflexia and spasticity. These complications make it difficult for patients to maintain a stable and comfortable posture, further complicating the use of preventive postural strategies.

Specialized equipment, such as pressure-relieving mattresses, cushions, and sliding surfaces, helps reduce pressure and friction [135]. Still, in SCI patients, microvascular dysfunction and endothelial impairment can limit the effectiveness of these interventions. Reduced blood flow and vascular narrowing contribute to tissue ischemia and necrosis, delaying wound healing and tissue regeneration [136].

Regular skin self-examinations are a key strategy for early detection of pressure points and erythema. Yet, sensory loss below the injury level prevents patients from feeling tissue damage, making it harder to detect problem areas in time. Additionally, prolonged suppression of neural activity during the acute phase, combined with the absence of protective reflexes, further weakens the body’s ability to prevent PIs.

In summary, while these therapeutic strategies are essential for PI prevention in SCI patients, the neurological and physiological complications of SCI make their effective implementation difficult. To improve prevention and treatment outcomes, healthcare professionals, caregivers, and family members must be aware of these challenges and adapt interventions to each patient’s specific needs.

#### 3.8.3. Moisture Management and Infection Control

In the context of SCI, moisture management and infection control present critical challenges due to the multisystem impairments these individuals experience. Neurological dysfunction following SCI affects not only mobility and sensation but also autonomic regulation across multiple systems, directly impacting sphincter control and skin barrier integrity. Urinary and fecal incontinence are common issues in this population and represent key risk factors for PIs [137]. Prolonged exposure to excessive moisture weakens the skin’s protective barrier, leading to maceration and increasing susceptibility to bacterial colonization [138]. Furthermore, autonomic nervous system dysfunction in patients with injuries above T6 exacerbates bladder and bowel control issues [3,16], worsening urinary retention, detrusor-sphincter dyssynergia, and impaired intestinal motility, all of which complicate effective moisture management strategies.

Implementing structured urinary continence protocols, including intermittent catheterization, is essential to minimizing prolonged skin exposure to urine, thereby reducing the risk of recurrent urinary tract infections, a frequent complication in SCI patients [139]. Additionally, external urine collectors, devices for urinary incontinence, and transanal irrigation systems for fecal incontinence can help control perineal skin moisture and reduce both mechanical and chemical irritation [140]. Still, even with these interventions, SCI patients remain highly vulnerable to skin breakdown due to compromised blood flow and altered tissue metabolism, both of which hinder skin resilience and healing.

Beyond moisture control, infection prevention is a top priority for these patients, as their compromised immune response and chronic inflammation increase susceptibility to bacterial colonization and systemic infections [93,94]. As previously discussed in The Immune System and Skin Microbiome, SCI-induced immune dysfunction, marked by excessive pro-inflammatory cytokines and reduced phagocytic activity [47,62,96], plays a critical role in delayed wound healing, particularly in PIs. While structured continence protocols and moisture management strategies help mitigate these risks [139,140], impaired blood flow and metabolic alterations in SCI patients make them particularly vulnerable to infection. The frequent presence of multidrug-resistant organisms (MDROs), including MRSA and Gram-negative bacteria, further complicates treatment [107], requiring close monitoring for early detection and timely intervention [117].

As with other aspects of SCI management, addressing moisture control and infection prevention requires a multidisciplinary approach that goes beyond preventing new PIs [141]. The ultimate goal is to ensure that wound closure and reconstructive surgery strategies are successful, preventing complications that could compromise the patient’s overall clinical outcome and long-term recovery [142].

### 3.9. Cell-Based Therapeutic Strategies in Regenerative Medicine

Extensive research has focused on preventing PIs, particularly in patients with limited mobility, such as those with SCI [9,135]. However, once these wounds become chronic, they are extremely challenging to manage, and conventional pharmacological and surgical approaches are not always sufficient [142,143]. In response to this growing need, tissue regeneration and reconstruction strategies using regenerative medicine and cell-based therapies have emerged as promising alternatives for enhancing wound healing and tissue repair [144,145].

Among the most explored approaches, mesenchymal stem cell (MSC) therapy has shown potential in promoting wound healing due to its regenerative capacity, paracrine activity, and immunomodulatory properties [146]. In both preclinical and clinical studies, MSCs have demonstrated efficacy in improving tissue repair in burns and chronic ulcers, indicating their potential applicability in SCI-related wounds [147,148,149]. Despite these promising findings, significant challenges remain in translating MSC-based therapies into effective clinical interventions.

One of the primary obstacles in SCI patients is the complex pathological environment in which MSCs must function. Chronic inflammation associated with advanced LPP, characterized by persistent activation of pro-inflammatory cytokines and oxidative stress, may hinder the regenerative potential of MSCs [115,150]. Additionally, vascular dysfunction, a key feature of SCI, impairs nutrient and oxygen delivery to the wound site, further limiting the effectiveness of MSCs therapy. Poor microvascularization remains a major barrier to wound healing, as adequate blood flow is crucial for tissue regeneration and graft survival [151,152,153].

Beyond physiological constraints, the long-term safety of MSC-based therapies remains a concern. While MSCs exhibit considerable regenerative potential, emerging evidence suggests their potential involvement in tumor development. MSCs have been shown to modulate the tumor microenvironment and support angiogenesis, raising concerns regarding their oncogenic potential [154,155]. These findings highlight the need for rigorous safety evaluations before MSCs therapies can be widely implemented in clinical settings.

Given the high prevalence of comorbidities in SCI patients, a standardized, one-size-fits-all approach is unlikely to be effective. Future research should focus on individualized therapeutic strategies that account for patient-specific factors such as immune status, metabolic alterations, and vascular health [156]. A deeper understanding of the molecular and cellular mechanisms underlying MSC behavior in SCI-associated wounds is essential to optimize these therapies for safe and effective clinical use [157].

## 4. Discussion

**Figure 1:**
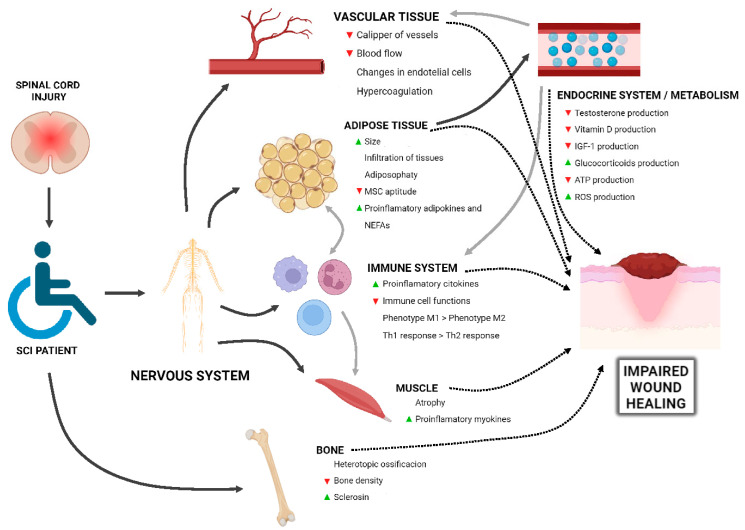
Overview of the physiological changes induced by spinal cord injury (SCI) that contribute to the development of chronic wounds, such as pressure injuries (PIs), and delayed healing. SCI disrupts multiple physiological systems, including vascular, adipose, immune, muscular, skeletal, and endocrine functions, triggering a cascade of effects that impair tissue repair. Black arrows indicate direct relationships, gray arrows represent indirect effects, and dashed arrows denote inhibitory interactions. ▲ Indicates an increase, while ▼ indicates a decrease in the respective parameter. Key pathological factors include reduced blood flow, immune dysregulation, muscle atrophy, and metabolic imbalances, all of which collectively contribute to impaired wound healing. References for each affected system: **Vascular tissue**: Vessel caliber [23,24,25], blood flow [23,25], endothelial changes [33], and hypercoagulability [47]; **Adipose tissue**: Size [23,24,25], infiltration of tissues [5,38,40,41,42,43], adiposophaty [36], mesenchymal stem cell (MSC) aptitude [49], and proinflammatory adipokines and NEFAs [5,38,40,41,42,43]; **Proinflammatory cytokines** [46,95,96,97], immune cell functions [94,98,99], phenotype M1 > phenotype M2 [60], and Th1 response > Th2 response [94]; **Muscle**: Atrophy [17,18,51] and proinflammatory myokines [59,60,61,62,63,64]. **Bone**: Heterotopic ossification [87,88], bone density [59,60,61,62,63,64], and sclerostin [63]; **Endocrine system/metabolism**: Testosterone production [57], vitamin D production [80], IGF-1 production [65,67], glucocorticoid production [91,94], ATP production [123], and ROS production [123]. The figure was created with BioRender.com.

Spinal cord injury extends beyond the damage of nervous system disruption to encompass a wide range of affected body structures and functions. Given the pathophysiology of SCI, PIs are the most prevalent chronic wounds observed in these patients. Wound healing is a complex process involving various factors and cellular elements that must coordinate to restore tissue integrity. While the main psychosocial and behavioral factors influencing PIs chronicity have been extensively described [10,11], we have analyzed the physiological changes that may not only lead to the occurrence of PIs in SCI but also delay their healing (Figure 1).

Janis and colleagues [132] described the four phases through which skin and underlying tissues pass until a wound resolves: hemostasis, inflammation mediated by immune cells, proliferation with new tissue growth, and maturation and remodeling, where tissue regains its pre-injury functionality. However, the alteration or excessive prolongation of any of these phases may delay tissue healing [10].

Chronic wounds exhibit marked differences compared to acute wounds, including increased metalloproteases, reduced number and functionality of keratinocytes and adipose tissue-resident stem cells (AT-MSCs), and variable presence of certain growth factors [158,159]. As per the evidence, there is a close relationship between loss of tissue blood flow due to prolonged pressure between two points and the occurrence of PIs. However, in SCI patients, comorbidities associated with SCI can further compromise circulation: autonomic dysreflexia [3], microcirculation alterations [34], and bone marrow failure [160], among others. On the other hand, glucose intolerance, dyslipidemia [122], or even microvascular dysfunction [35] are notable factors that may contribute to and promote the development of cardiovascular diseases. Furthermore, immobility in these individuals leads to the formation of constant pressure areas, which can exacerbate the previously described changes in body composition (increased adiposity, changes in bone morphology), contributing to peripheral tissue ischemia.

Moreover, nutrient and oxygen supply, as well as waste removal, are vital for granulation tissue formation. Additionally, the formation of new blood vessels is necessary to accelerate the process. Given the lower availability and consumption of oxygen, along with poorer mitochondrial function in these patients, tissues opt for the use of anaerobic pathways, resulting in increased production of free radicals and reinforcing the local inflammatory environment [124]. Cytokines such as IL-1, IL-6, and TNF-α, among others, also contribute to prolonging this condition [47]. The presence of these cytokines, as well as ROS, prevents macrophages from transitioning to an M2 phenotype, hindering PIs healing [62]. Such polarization toward the M1 phenotype and its negative role has been previously described in other tissues [161,162,163].

Infectious complications of PIs also pose an additional challenge in SCI patients. Immobility, immunosuppression, alterations in local circulation, or the generation of moist areas, closely related to incontinence underlying the injury, all accompanied by inadequate care, are factors that can contribute to wound infection in this patient group. In this context, it is crucial to assess the skin and periulcer tissue status before performing any surgical intervention aimed at PIs reconstruction, as high bacterial loads eventually generate a biofilm that favors an optimal environment for microorganisms [8], evading immune system action while sustaining a proinflammatory environment associated with delayed healing, increased associated complications, and possible recurrence of the wound [164,165]. Furthermore, poor perfusion, hypoxia, and ROS generation, previously described for their involvement in SCI, have also been associated with increased multi-genus bacterial colonization [166]. However, as mentioned in the review, not only bacterial infections are implicated in poorer surgical recovery. Specifically, our group detected delayed PIs healing in SCI patients who had previously experienced SARS-CoV-2 infection before restorative surgery, correlating the chronic phase of the pathology with specific cellular and molecular markers [117]. Given this, and considering that SCI is a chronic pathology that promotes the development of comorbidities compromising both the formation and healing of PIs, further studies are needed to explore alternative therapeutic strategies, including cell-based approaches while taking into account the multiple pathophysiological factors associated with SCI. These efforts will be essential to improving both the prevention and treatment of chronic wounds in such a vulnerable population.

## 5. Conclusions

To end up, SCI predisposes patients to the development of PIs and complicates their recovery. In addition to the new behavioral habits generated by this new condition, a multitude of changes occur at the tissue and molecular level. The present study aims to clarify these changes to enhance the understanding of the pathophysiology of chronic wounds and thereby facilitate the development of new therapies aimed at their healing.

## Figures and Tables

**Table 1:** Overview of the most relevant pathophysiological changes involved in the development and progression of PIs. ↑ indicates an increase, while ↓ indicates a decrease.

System/Tissue	Pathophysiological Changes	Consequences in Pressure Injuries
Vascular	↓ Vascular caliber and blood flow. Microcirculation alterations and endothelial dysfunction. Generation of reactive oxygen species (ROS) and soluble tumor necrosis factor-alpha (sTNFα).	Tissue ischemia and necrosis. Chronic hypoxia impairing cell regeneration. Cellular damage due to oxidative stress.
Adipose Tissue	↑ Total adiposity, especially visceral fat. Elevated TNF and IL-6 production. Tissue hypoxia due to low capillary density. ↑ Fatty acid-binding protein (FABP).	Decreased mesenchymal stem cell functionality. Ischemia and increased susceptibility to tissue necrosis. Chronic inflammatory response interfering with healing.
Muscle Tissue	↓ Muscle mass and ↑ intramuscular fat. Conversion of type I fibers to type IIb. ↓ IGF-1 and ↑ inflammatory mediators.	Reduced protection over bony prominences. Greater tissue deformation under pressure. Increased risk of deep ulcers and poor healing.
Bone Tissue	↓ Bone mineral density (osteoporosis). Formation of heterotopic ossification. ↓ Vitamin D and osteocalcin.	Increased risk of fractures and additional pressure points. Reduced bone resistance and structural support. Greater susceptibility to pressure ulcers over bony prominences.
Immune System and Microbiota	Chronic inflammatory state (↑ IL-6, TNF-α). Immune suppression (↓ neutrophils, monocytes, NK cells). Alterations in skin microbiota (Gram-negative bacteria, antibiotic resistance). Lipopolysaccharides link to MSCs.	Inhibited macrophage transition to M2 phenotype, delaying healing. Increased susceptibility to infections. Bacterial colonization of wounds and biofilm formation. MSCs differentiation toward heterotopic ossification.
Endocrine and Metabolism	Insulin resistance. ↓ IGF-1 and testosterone production. Decreased basal energy expenditure and lipid metabolism alterations. Changes to pathological lipid and cholesterol profile.	Impaired tissue regeneration. Predisposition to obesity and metabolic syndrome. Increased proinflammatory adipokines, worsening inflammation. Development of hypertension and atherosclerosis.

## Data Availability

No new data were created or analyzed in this study. Data sharing is not applicable to this article.
